# A new leaf essential oil from the Andean species *Gynoxys szyszylowiczii* Hieron. of southern Ecuador: chemical and enantioselective analyses

**DOI:** 10.1038/s41598-024-67482-z

**Published:** 2024-07-16

**Authors:** Yessenia E. Maldonado, Omar Malagón, Nixon Cumbicus, Gianluca Gilardoni

**Affiliations:** 1 Departamento de Química, Universidad Técnica Particular de Loja (UTPL), Calle Marcelino Champagnat s/n, 110107 Loja, Ecuador; 2 Departamento de Ciencias Biológicas y Agropecuarias, Universidad Técnica Particular de Loja (UTPL), Calle Marcelino Champagnat s/n, Loja, 110107 Ecuador

**Keywords:** Mass spectrometry, Stereochemistry, Secondary metabolism

## Abstract

The essential oil obtained from the dry leaves of *Gynoxys szyszylowiczii* Hieron. was described in this study for the first time. The chemical analysis, conducted on two stationary phases of different polarity, permitted to identify sixty-four compounds, that were quantified with at least one column. The main components, on a non-polar and polar stationary phase respectively, were germacrene D (21.6–19.2%), α-pinene (4.4–4.9%), *n*-tricosane (4.3% on both columns), (*E*)-β-caryophyllene (3.3–4.3%), 1-docosene (3.2–2.8%), α-cadinol (2.8–3.1%), and *cis*-β-guaiene (2.6–3.0%). This investigation was complemented by the enantioselective analysis of some major chiral compounds, carried out on two chiral selectors based on β-cyclodextrines. As a result, (*S*)-( +)-α-phellandrene, (*S*)-( +)-β-phellandrene, and (1*S*,2*R*,6*R*,7*R*,8*R*)-( +)-α-copaene appeared enantiomerically pure, whereas α-pinene, β-pinene, terpinen-4-ol, and germacrene D were detected as scalemic mixtures. Finally, linalool was practically racemic. The distillation yield, analytically calculated by weight of dry plant material, was 0.03%.

## Introduction

During the last decades, the increasing and almost exhaustive information about phytochemistry in the boreal hemisphere, shifted the interest of natural product chemists from temperate countries to tropical regions, where most of the botanical species are often still unstudied. In this regard, Ecuador is an ideal place to investigate secondary metabolites since it has been mentioned by the United Nations among the seventeen “megadiverse” countries^1^. So far, mainly for historical reasons, Ecuador’s outstanding biodiversity has been very poorly investigated, and most of its native and endemic plants still lack a systematic chemical description^2,3^. For this reason, and with the aim of contributing to the chemotaxonomic description and the biological enhancement of the Ecuadorian biodiversity, our group has been searching for new natural products in this region during the last twenty years^4,5^. More recently, our interest focused on the description of new essential oils (EOs), with emphasis on their chemical compositions, enantioselective analyses, and gas chromatography–olfactometry (GC-O) profiles^6–9^. In particular, the present research is a part of an unfunded project, dealing on the systematic description of volatile fractions from the main species of genus *Gynoxys* Cass. (Asteraceae) in southern Ecuador. In this respect, based on their accessibility on the territory, twelve unprecedented species were selected for their EOs to be described: *Gynoxys miniphylla* Cuatrec., *Gynoxys rugulosa* Muschl., *Gynoxys buxifolia* (Kunth) Cass., *Gynoxys laurifolia* (Kunth) Cass., *Gynoxys szyszylowiczii* Hieron., *Gynoxys sancti-antonii* Cuatrec., *Gynoxys calyculisolvens* Hieron., *Gynoxys pulchella* (Kunth) Cass., *Gynoxys cuicochensis* Cuatrec., *Gynoxys reinaldii* Cuatrec., *Gynoxys hallii* Hieron., and *Gynoxys azuayensis* Cuatrec. All these species grow wild in the highlands of the province of Loja, and they are native or endemic of this region. So far, the chemical and enantioselective analyses of the EOs from *G. miniphylla*, *G. rugulosa*, *G. buxifolia*, and *G. laurifolia* have been published, *G. szyszylowiczii* is the object of the present study, whereas all the other species are currently under investigation^10–13^. The species *Gynoxys szyszylowiczii* Hieron. is a quite uncommon taxon, with no botanical synonyms and it is absent in the Catalogue of Vascular Plants of Ecuador^14–16^. However, despite the diffusion area of this species corresponds to Peru, the only available reference specimen was collected in Ecuador in 1998, in the same collection place of the samples that were used in this research (see plant material, Section "Plant material")^15^. Botanically, *G. szyszylowiczii* is a tree, 5 m tall, with hexagonal branches when young. The plant is densely reddish-tomentose, with opposite petiolate leaves. Blades are elliptical-oblong or ovate-oblong, with rounded or slightly cordate base, obtuse and pointed apex, denticulate margin, subcoriaceous, sparsely tomentose above when young. The inflorescences are corymbose and branched^17^.

No traditional use has been reported for this species in Ecuador or elsewhere. To the best of the authors’ knowledge, this report represents the first description of an EO obtained from *G. szyszylowiczii*. The study has been completed with the enantioselective analysis of some major chiral terpenes and terpenoids.

## Results

### Chemical analysis

**Figure 1:**
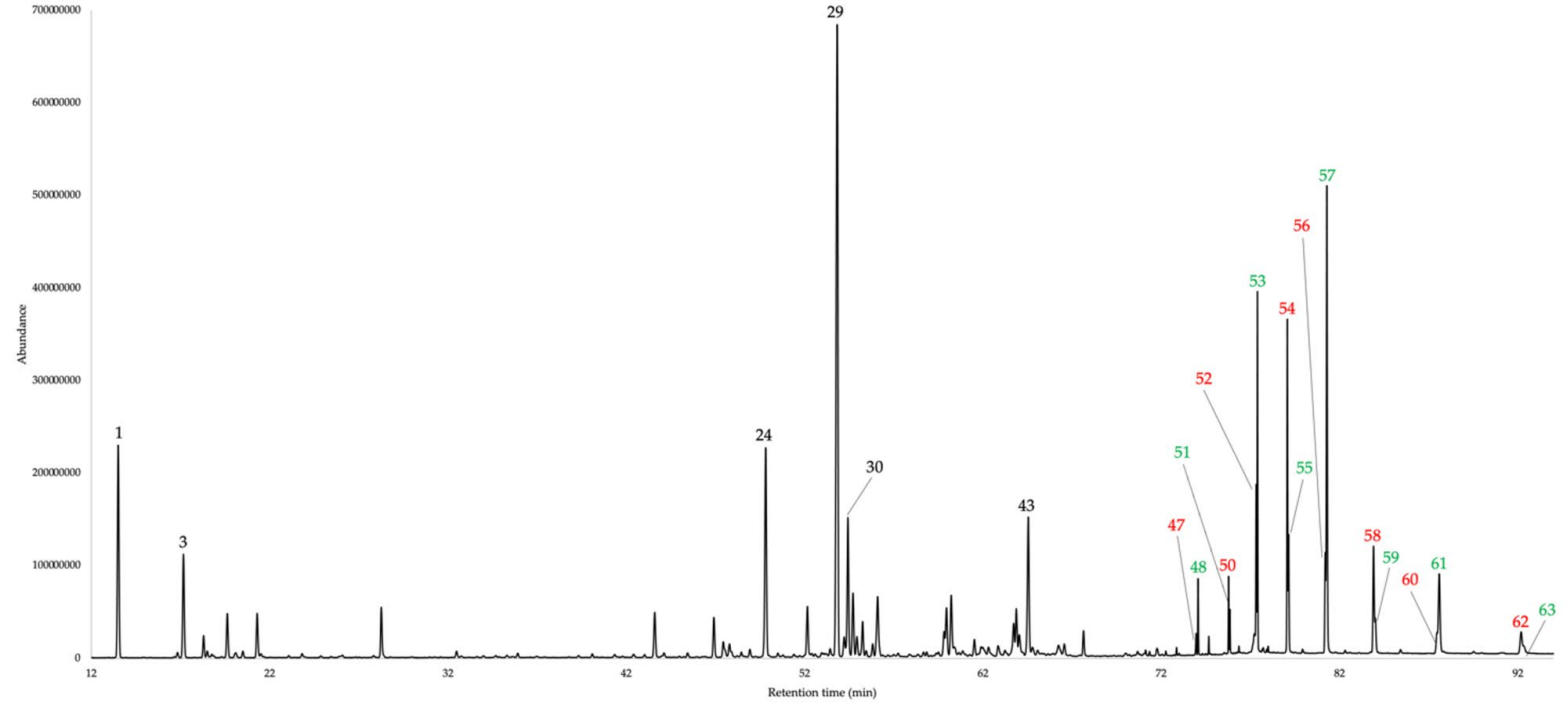
GC–MS profile of *G. szyszylowiczii* EO on a 5%-phenyl-methylpolysiloxane stationary phase. The peak numbers refer to Table 1. Black numbers: major terpenes and terpenoids (≥ 3.0%); green numbers: heavy *n*-alkanes; red numbers: heavy 1-alkenes.

**Figure 2:**
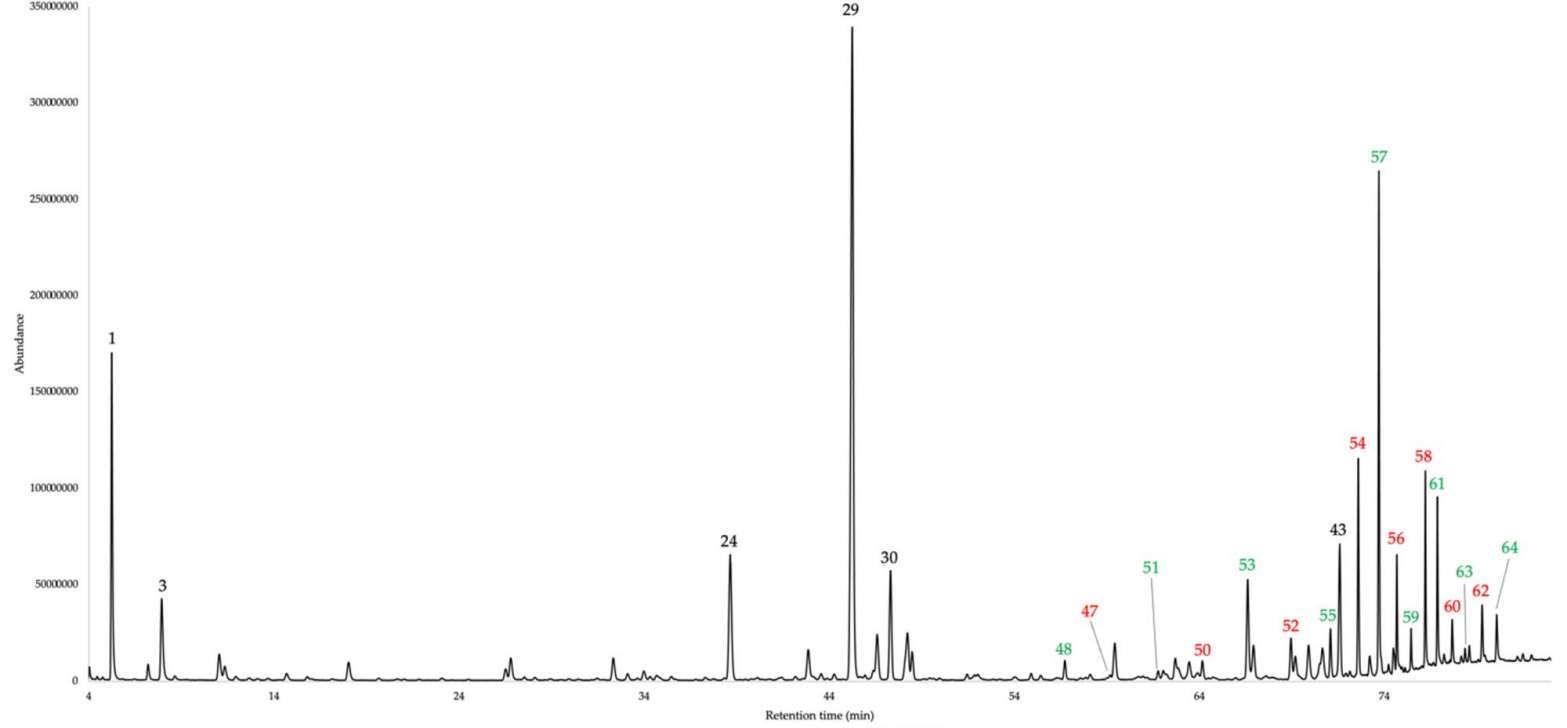
GC–MS profile of *G. szyszylowiczii* EO on a polyethylene glycol stationary phase. The peak numbers refer to Table 1. Black numbers: major terpenes and terpenoids (≥ 3.0%); green numbers: heavy *n*-alkanes; red numbers: heavy 1-alkenes.

The dry leaves of *G. szyszylowiczii* analytically produced an EO with a 0.03 ± 0.004% yield (w/w), as a mean value over four distillations. The volatile fraction, despite in cyclohexane solution, was characterized by a mild sweet, hay like odour, very similar to the ones of most of the *Gynoxys* EOs so far studied. The qualitative analysis permitted to identify and quantify sixty-four components with at least one of the two columns used. The quantification corresponded to 86.1–84.1% of the whole oil mass, on the non-polar and polar stationary phases respectively. The EO was dominated by the sesquiterpene fraction, with sesquiterpenes and oxygenated sesquiterpenoids accounting for 37.5–35.6% and 10.8–10.0% of the total mass respectively. Secondly, as usual in *Gynoxys* EOs, a heavy aliphatic fraction is present, corresponding to 27.5–28.5% of the entire oil composition. Finally, a monoterpene fraction was identified, with monoterpenes and oxygenated monoterpenoids corresponding together to 10.1–9.8% of the whole volatile fraction. Major constituents of *G. szyszylowiczii* EO (≥ 3.0% on at least one column) were germacrene D (21.6–19.2%, peak 29), α-pinene (4.4–4.9%, peak 1), *n*-tricosane (4.3% on both columns, peak 57), (*E*)-β-caryophyllene (3.3–4.3%, peak 24), 1-docosene (3.2–2.8%, peak 54), α-cadinol (2.8–3.1%, peak 43), and *cis*-β-guaiene (2.6–3.0%, peak 30). The complete chemical analysis is reported in Table 1, whereas the gas chromatographic profiles with both stationary phases are reported in Figs. 1 and 2.

**Table 1:** Qualitative (GC–MS) and quantitative (GC-FID) chemical composition of *G. szyszylowiczii* EO on 5%-phenyl-methylpolysiloxane and polyethylene glycol stationary phases.

N	Compounds	5%-phenyl-methylpolysiloxane					polyethylene glycol				
		LRI^a^	LRI^b^	%	σ	Reference	LRI^a^	LRI^b^	%	σ	Reference
1	α-Pinene	933	932	4.4	1.62	^18^	1016	1017	4.9	1.05	^19^
2	Sabinene	975	969	trace	–	^18^	1114	1112	0.1	0.03	^19^
3	β-Pinene	979	974	2.4	0.45	^18^	1102	1097	2.3	0.38	^19^
4	Myrcene	993	988	0.6	0.17	^18^	1159	1157	0.5	0.11	^19^
5	2-Pentyl furan	995	984	0.3	0.05	^18^	1229	1229	0.1	0.02	^20^
6	6-Methyl-5-hepten-2-one	999	981	0.1	0.01	^18^	1334	1335	0.1	0.01	^21^
7	α-Phellandrene	1009	1002	1.2	0.22	^18^	1154	1158	0.8	0.16	^19^
8	(2*E*,4*E*)-Heptadienal	1014	1005	0.1	0.05	^18^	1459	1454	0.2	0.04	^19^
9	α-Terpinene	1020	1014	0.4	0.04	^18^	1169	1167	0.1	0.03	^19^
10	*p*-Cymene	1030	1020	0.8	0.14	^18^	1261	1265	0.6	0.13	^22^
11	Limonene	1032	1024	0.1	0.04	^18^	1189	1191	0.1	0.01	^19^
12	β-Phellandrene	1034	1025	0.1	0.02	^18^	1199	1195	0.3	0.03	^19^
13	(*E*)-β-Ocimene	1051	1044	0.1	0.03	^18^	1248	1244	0.1	0.01	^19^
14	1-Octanol	1086	1063	0.1	0.01	^18^	1559	1558	0.1	0.01	^20^
15	Linalool	1110	1096	0.1	0.02	^18^	1551	1554	0.1	0.01	^22^
16	*n*-Nonanal	1116	1100	1.3	0.10	^18^	1388	1389	0.9	0.06	^23^
17	Terpinen-4-ol	1191	1174	0.1	0.02	^18^	1592	1591	0.1	0.01	^24^
18	(2*E*,4*Z*)-Decadienal	1309	1292	0.1	0.01	^18^	1755	1752	0.1	0.01	^25^
19	*p*-Vinyl guaiacol	1327	1309	1.9	0.25	^18^	2187	2187	1.7	0.05	^26^
20	(2*E*,4*E*)-Decadienal	1335	1315	0.3	0.02	^18^	1797	1780	0.1	0.10	^27^
21	α-Copaene	1377	1374	1.1	0.06	^18^	1472	1472	0.8	0.03	^24^
22	β-Bourbonene	1385	1387	0.7	0.25	^18^	1498	1502	0.4	0.07	^19^
23	Longifolene	1407	1407	0.8	0.21	^18^	1620	1623	0.7	0.20	^28^
24	(*E*)-β-Caryophyllene	1421	1417	3.3	1.67	^18^	1574	1575	4.3	0.35	^29^
25	(*E*)-β-Farnesene	1456	1454	1.4	0.15	^18^	1662	1660	0.1	0.01	^19^
26	α-Humulene	1459	1452			^18^	1645	1650	1.2	0.10	^19^
27	9-*epi*-(*E*)-Caryophyllene	1463	1464	0.1	0.01	^18^	1574	1572	0.1	0.01	^30^
28	4,5-di-*epi*-Aristolochene	1473	1471	0.4	0.01	^18^	1770	–	0.1	0.01	§
29	Germacrene D	1485	1480	21.6	1.86	^18^	1686	1690	19.2	2.05	^19^
30	*cis*-β-Guaiene	1491	1492	2.6	0.98	^18^	1669	1667	3.0	1.01	^31^
31	α-Zingiberene	1495	1493	0.4	0.06	^18^	1723	1721	0.3	0.05	^32^
32	Bicyclogermacrene	1500	1500	1.4	0.25	^18^	1710	1706	1.0	0.38	^33^
33	α-Muurolene	1503	1500			^18^	1706	1706	0.3	0.04	^34^
34	(*E*,*E*)-α-Farnesene	1509	1505	1.9	0.34	^18^	1744	1740	4.1	1.35	^19^
35	γ-Cadinene	1518	1513	0.3	0.05	^18^	1737	1738			^35^
36	δ-Cadinene	1523	1522	1.5	0.28	^18^	1739	1738			^36^
37	Germacrene D-4-ol	1586	1574	0.8	0.33	^18^	2032	2038	0.9	0.15	^37^
38	Spathulenol	1588	1577	1.4	0.50	^18^	2104	2106	1.3	0.07	^35^
39	Caryophyllene oxide	1592	1582	1.8	0.47	^18^	1951	1953	1.3	0.08	^38^
40	Ledol	1615	1602	0.7	0.14	^18^	2003	2007	0.4	0.06	^39^
41	*epi*-α-Cadinol	1654	1638	1.0	0.12	^18^	2155	2154	0.8	0.04	^40^
42	*epi*-α-Muurolol	1657	1640	1.5	0.35	^18^	2171	2171	1.6	0.40	^31^
43	α-Cadinol	1669	1652	2.8	0.56	^18^	2216	2221	3.1	0.06	^39^
44	Amorpha-4,9-dien-2-ol	1705	1700	0.6	0.06	^18^	2345	–	0.4	0.06	§
45	*n*-Pentadecanal	1727	1724	0.7	0.05	^18^	2020	2020	0.7	0.07	^41^
46	14-Hydroxy-δ-cadinene	1808	1803	0.2	0.07	^18^	2587	2607	0.2	0.05	^36^
47	1-Nonadecene	1895	1895	0.3	0.04	^42^	1946	1943	0.5	0.05	^43^
48	*n*-Nonadecane	1900	1900	0.7	0.06	^18^	1900	1900	0.6	0.06	–
49	*n*-Heptadecanal	1933	1930	0.2	0.01	^44^	2238	2247	0.5	0.03	^45^
50	1-Eicosene	1995	1993	0.7	0.07	^42^	2047	2047	0.9	0.08	^46^
51	*n*-Eicosane	2000	2000	0.4	0.04	^18^	2000	2000	0.3	0.03	–
52	1-Heneicosene	2095	2094	1.6	0.16	^47^	2150	2167	1.4	0.13	^46^
53	*n*-Heneicosane	2100	2100	2.7	0.18	^18^	2100	2100	2.5	0.22	–
54	1-Docosene	2195	2196	3.2	0.79	^42^	2256	–	2.8	0.29	§
55	*n*-Docosane	2200	2200	1.1	0.10	^18^	2200	2200	1.4	0.24	–
56	1-Tricosene	2296	2294	2.2	0.27	^47^	2356	–	1.8	0.13	§
57	*n*-Tricosane	2300	2300	4.3	1.19	^18^	2300	2300	4.3	0.24	–
58	1-Tetracosene	2396	2396	1.5	0.34	^42^	2454	–	1.8	0.16	§
59	*n*-Tetracosane	2400	2400	0.4	0.08	^18^	2400	2400	0.5	0.05	–
60	1-Pentacosene	2496	2496	0.3	0.21	^42^	2553	–	0.7	0.07	§
61	*n*-Pentacosane	2500	2500	1.1	0.54	^18^	2500	2500	1.5	0.49	–
62	1-Hexacosene	2597	2596	1.5	0.99	^48^	2650	–	1.4	0.40	§
63	*n*-Hexacosane	2600	2600	0.4	0.06	^18^	2600	2600	0.3	0.05	–
64	*n*-Heptacosane	–	–	–	–		2700	2700	1.3	0.50	–
	Monoterpenes			10.1					9.8		
	Oxygenated monoterpenoids			0.2					0.2		
	Sesquiterpenes			37.5					35.6		
	Oxygenated sesquterpenoids			10.8					10.0		
	Others			27.5					28.5		
	Total			86.1					84.1		

^a^calculated linear retention index (LRI); ^b^ reference linear retention index (LRI); % = percent by weight of EO; σ = standard deviation; § = identification by MS only.

### Enantioselective analysis

The enantioselective analysis permitted to determine the distribution and enantiomeric excess (ee) of height chiral terpenes. Among them, (*S*)-( +)-α-phellandrene, (*S*)-( +)-β-phellandrene, and (1*S*,2*R*,6*R*,7*R*,8*R*)-( +)-α-copaene were enantiomerically pure, linalool was practically racemic, whereas α-pinene, terpinen-4-ol, and germacrene D were present as scalemic mixtures. The analysis was based on two enantioselective columns to ensure the enantiomer separation of all the detected chiral compounds. As usual, the enantioselective analysis is limited to the chiral components for which enantiomerically pure standards were available. The results of the enantioselective analysis are detailed in Table 2.

**Table 2:** Enantioselective analysis of some chiral terpenes from *G. szyszylowiczii* EO.

Enantiomers	2,3-diethyl-6-*tert*-butyldimethylsilyl-β-cyclodextrin			2,3-diacethyl-6-*tert*-butyldimethylsilyl-β-cyclodextrin		
	LRI	Composition (%)	ee (%)	LRI	Composition (%)	ee (%)
(1*S*,5*S*)-(-)-α-pinene		Unresolved		925	23.9^§^	52.2
(1*R*,5*R*)-( +)-α-pinene				927	76.1^§^	
(1*S*,5*S*)-(-)-β-pinene	960	5.7	88.7		Unresolved	
(1*R*,5*R*)-( +)-β-pinene	949	94.3				
(*S*)-( +)-α-phellandrene	1023	100.0	100.0	1022	100.0	100.0
(*R*)-(-)-α-phellandrene	1020	–		1024	–	
(*S*)-( +)-β-phellandrene	1062	100.0	100.0	1071	100.0	100.0
(*R*)-(-)-β-phellandrene	1051	–		1073	–	
(*R*)-(-)-linalool	1182	49.1	1.8	1274	48.7	2.6
(*S*)-( +)-linalool	1195	50.9		1280	51.3	
(1*R*,2*S*,6*S*,7*S*,8*S*)-(-)-α-copaene	1320	–	100.0		Unresolved	
(1*S*,2*R*,6*R*,7*R*,8*R*)-( +)-α-copaene	1323	100.0				
(*R*)-(-)-terpinen-4-ol		Unresolved		1293	42.2*	15.6
(*S*)-( +)-terpinen-4-ol				1297	57.8*	
(*R*)-( +)-germacrene D	1466	98.8	97.6		Unresolved	
(*S*)-(-)-germacrene D	1472	1.2				

LRI = calculated linear retention index; ee = enantiomeric excess; ^§^ partially resolved peaks; * extracting ion 111 m*/z.*

## Discussion

**Figure 3:**
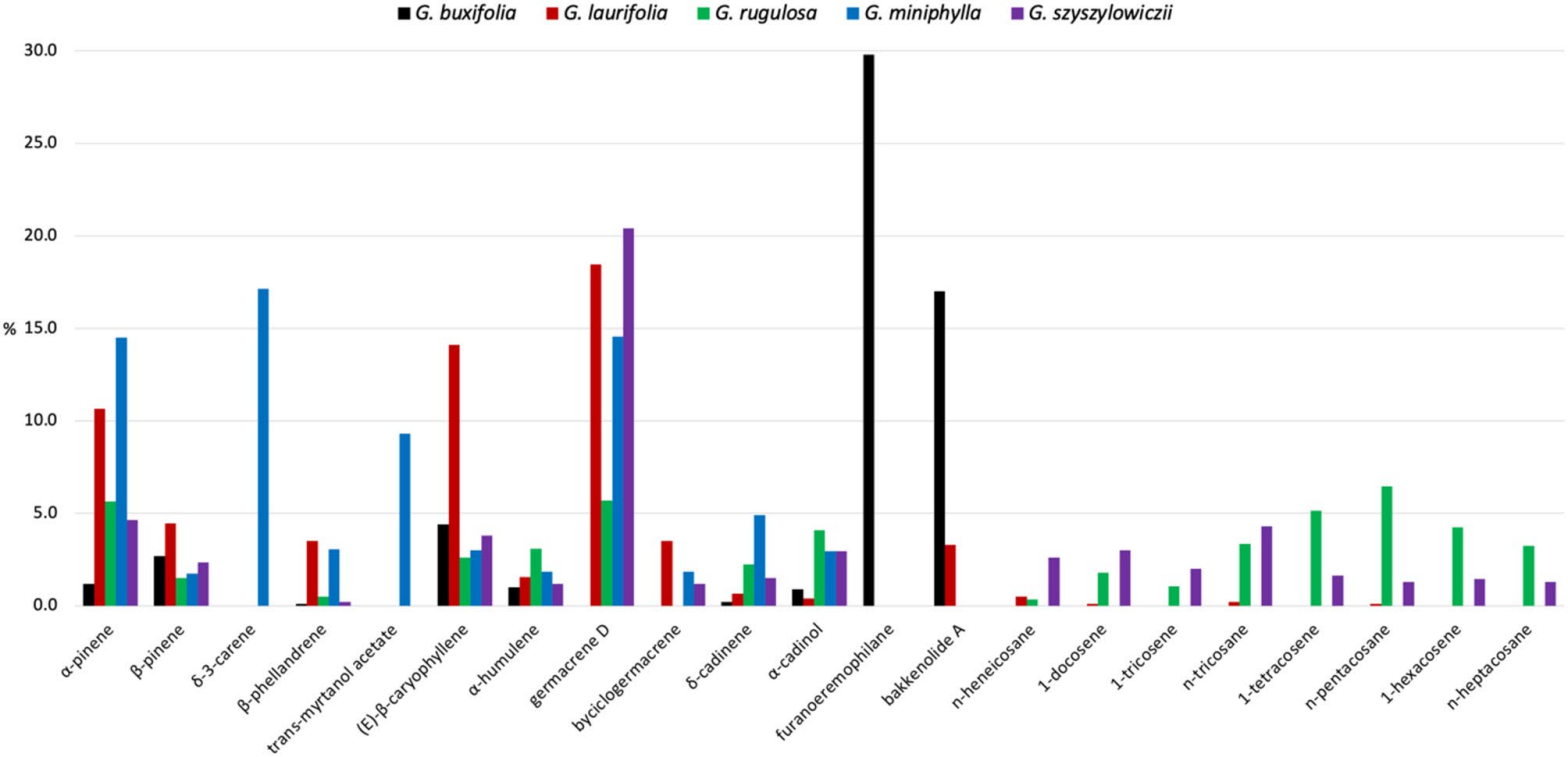
Percent abundance of major components in *G. buxifolia*, *G. laurifolia*, *G. rugulosa*, *G. miniphylla*, and *G. szyszylowiczii* EOs. The values correspond to constituents whose abundance is ≥ 3.0% in at least one plant EO, as a mean value between the two GC columns.

The EO from the leaves of *G. szyszylowiczii* presented a GC pattern similar to the one of other already known *Gynoxys* EOs^10–13^. In fact, we can observe in Figs. 1 and 2, that three main fractions were evident: a minoritarian monoterpene fraction, a majoritarian sesquiterpene fraction, and an important heavy aliphatic fraction. The compared chemical compositions of major components in *G. buxifolia*, *G. laurifolia*, *G. rugulosa*, *G. miniphylla*, and *G. szyszylowiczii* EOs are plot in Fig. 3 where compounds, whose abundance is ≥ 3.0% in at least one EO, are represented.

According to Fig. 3, it can be observed that *G. buxifolia* EO was completely different from the others, from both the qualitative and quantitative points of view. On the other hand, *G. laurifolia*, *G. rugulosa*, *G. miniphylla*, and *G. szyszylowiczii* produced more similar volatile fractions. In fact, in all the species other than *G. buxifolia*, germacrene D, α-pinene, (*E*)-β-caryophyllene, β-pinene, α-humulene, δ-cadinene, and α-cadinol were main components, common to all the EOs in a quite similar amount. For what concerns the heavy aliphatic fraction, *G. szyszylowiczii* was similar to *G. rugulosa*, despite the relative abundances differed. Only in *G. miniphylla* EO, δ-3-carene and *trans*-myrtanol acetate were among the main compounds. The potential anti-inflammatory and cholinergic activities of these EOs, among many others based on their chemical analyses and according to literature, were discussed in our previous studies. These properties could speculatively be extended also to *G. szyszylowiczii*, due to their similar compositions^11,13^. Finally, the enantioselective analysis of and *G. szyszylowiczii* EO can also be compared with those conducted on *G. buxifolia*, *G. laurifolia*, *G. rugulosa*, and *G. miniphylla*, as indicated in Table 3.

**Table 3:** Compared enantiomeric distributions of some chiral compounds in the EOs of *G. buxifolia*, *G. laurifolia*, *G. rugulosa*, *G. miniphylla*, and *G. szyszylowiczii*.

Enantiomers	Enantiomeric distribution (%)				
	*G. buxifolia*	*G. laurifolia*	*G. rugulosa*	*G. miniphylla*	*G. szyszylowiczii*
(1*S*,5*S*)-(-)-α-pinene	100.0	64.8	62.9	99.1	23.9
(1*R*,5*R*)-( +)-α-pinene	–	35.2	37.1	0.9	76.1
(1S,5S)-(-)-β-pinene	100.0	100.0	–	55.9	5.7
(1*R*,5*R*)-( +)-β-pinene	–	–	100.0	44.1	94.3
(1*S*,5*S*)-( −)-sabinene	84.6	50.2	27.8	43.0	–
(1*R*,5*R*)-( +)-sabinene	15.4	49.8	72.2	57.0	–
(*R*)-( −)-α-phellandrene	–	100.0	40.9	100.0	–
(*S*)-( +)-α-phellandrene	100.0	–	59.1	–	100.0
(*R*)-(-)-β-phellandrene	–	100.0	–	100.0	–
(*S*)-( +)-β-phellandrene	100.0	–	100.0	–	100.0
(*R*)-(-)-linalyl acetate	–	–	–	–	–
(*S*)-( +)-linalyl acetate	–	100.0	–	–	–
(*R*)-(-)-linalool	–	62.3	52.4	–	48.9
(*S*)-( +)-linalool	–	37.7	47.6	–	51.1
(1*R*,2*S*,6*S*,7*S*,8*S*)-(-)-α-copaene	–	–	4.3	–	–
(1*S*,2*R*,6*R*,7*R*,8*R*)-( +)-α-copaene	–	–	95.7	–	100.0
(*R*)-(-)-terpinen-4-ol	–	53.0	42.6	–	42.2
(*S*)-( +)-terpinen-4-ol	100.0	47.0	57.4	–	57.8
(*S*)-( −)-α-terpineol	–	66.3	50.1	–	–
(*R*)-( +)-α-terpineol	–	33.7	49.9	–	–
(*S*)-(-)-germacrene D	–	100.0	4.5	95.5	1.2
(*R*)-( +)-germacrene D	–	–	95.5	4.5	98.8

As we can observe, the EOs did not apparently shared a common distribution pattern. In fact, in different volatile fractions, different enantiomers of the same compound could be detected in an enantiomerically pure form. In other cases, where scalemic mixtures were observed, the dominant enantiomer was not always the same. On the other hand, β-phellandrene always appeared as a pure enantiomer, whereas β-pinene, sabinene, α-phellandrene, linalool, terpinen-4-ol, and α-terpineol were sometimes observed as almost racemic. As usual, the presence of different enantiomers in a natural source can be justified by the different biological properties that they exert in living organisms, due to the chiral character of enzymes and receptors^49,50^. About *G. szyszylowiczii* EO, we could observe the presence of α-copaene as an abundant compound (about 1%). This sesquiterpene is not common in this botanical genus and has been observed so far only in *G. rugulosa*, where it is present in his almost enantiomerically pure dextrorotatory form. This enantiomer has been described for being a chemical rendezvous cue for the Mediterranean fruit fly *Ceratitis capitata*^51^. Furthermore, according to literature, (1*S*, 2*R*, 6*R*, 7*R*, 8*R*)-( +)-α-copaene affected *C. capitata* virgin females, inducing “pseudomale” courtship behaviour in a short-range bioassay.

Another issue is the different enantiomeric excess of α-pinene and β-pinene within the same species (see Table 3). In fact, both α-pinene and β-pinene derive from the same pinyl cation, by effect of the same enzyme pinene synthase^52^. Since the final stereochemistry of pinenes depends on the configuration of the common carbocation, the same enantiomeric excess is expected to be observed, for the same enantiomer, in both pinenes. However, a different experimental evidence is reported in the present study. This phenomenon could theoretically be explained, for example through the enantiospecific transformation of only one enantiomer into another unidentified compound.

## Methods

### Plant material

The leaves of *G. szyszylowiczii* were collected on 9 March 2020 in the province of Loja (Ecuador), from different trees and treelets located within the distance of 200 m from a central point, whose coordinates were 03° 40′ 52'' S and 79° 15′ 57'' W. The taxonomic identification was carried out by one of the authors (N.C.), and a botanical specimen is conserved at the herbarium of the Universidad Técnica Particular de Loja (HUTPL), with voucher 14,670. The plant was collected at the same collection place of a reference specimen, conserved at the Missouri Botanical Garden (St. Louis, MO, USA) with code 5,167,495, that was used for identification. After collection, the plant material was dried at 35 °C for 48 h and stored in a fresh dark place until use. Both collection and investigation were conducted under permission of the Ministry of Environment, Water and Ecological Transition of Ecuador, as reported in the policy statement about plant investigation.

### Distillation and sample preparation

Four amounts of dry leaves (80.7 g, 81.5 g, 80.7 g, and 80.8 g respectively) were analytically steam distilled for four hours each, as previously described in literature, in a modified Dean-Stark apparatus^11^. The volatile fraction, after condensation, was extracted in situ through 2 mL of cyclohexane, previously spiked with *n*-nonane as internal standard (0.7 mg/mL). An internal standard was necessary because the EO was obtained as a solvent solution of unknown concentration. The four repetitions, obtained in this way, were directly injectable into GC and they were stored at -15 °C until use. Both cyclohexane and *n*-nonane were analytical grade and purchased from Sigma-Aldrich (St. Louis, MO, USA).

### Qualitative (GC–MS) and quantitative (GC-FID) chemical analyses

Both qualitative and quantitative analyses were conducted on two different columns (30 m long, 0.25 mm internal diameter and 0.25 μm film thickness), purchased from Agilent Technology (Santa Clara, CA, USA). One column was based on 5%-phenyl-methylpolysiloxane (HP-5) as a non-polar stationary phase and the other one was based on polyethylene glycol (HP-INNOWax) as a polar stationary phase. For the qualitative analysis, the columns were installed in a gas chromatograph (GC) model Trace 1310, coupled to a simple quadrupole detector (MS) model ISQ 7000 (Thermo Fisher Scientific, Walthan, MA, USA). The ion source was an electron ionization (EI) device, with 70 eV ionization energy. The MS was operated in SCAN mode, with the mass analyser set at the mass range of 40–400 m*/z*. Both ion source and transfer line were heated at 230 °C. For the quantitative analysis, the same GC as the qualitative one was used. However, a flame ionization detector (FID), set at 230 °C, was employed instead of MS. In both qualitative and quantitative analyses, the injection volume (1 μL), injector conditions, carrier gas, and thermal program were the same. The injector was operated in split mode (40:1 for GC–MS and 10:1 for GC-FID) and it was heated at 230 °C, whereas the carrier gas was helium (purchased from Indura, Guayaquil, Ecuador), that was set at the constant flow of 1 mL/min. In the qualitative analysis, the EO components were identified by comparison of each MS spectrum and linear retention index (LRI) with data from literature (see Table 1). The LRIs were calculated according to Van den Dool and Kratz, based on a mixture of homologues *n*-alkanes in the range C_9_–C_27_ (Sigma-Aldrich, St. Louis, MO, USA)^53^. In the quantitative analysis, each constituent was quantified through a six-point calibration curve, using *n*-nonane (Sigma-Aldrich, St. Louis, MO, USA) as internal standard and isopropyl caproate as calibration standard, as previously described in literature^54^. Isopropyl caproate was synthetised in one of the authors’ laboratories (G.G.) and purified until 98.8% (GC-FID purity). Both calibration curves afforded a correlation coefficient of 0.999. The EO components were quantified calculating each relative response factor *versus* isopropyl caproate, based on its combustion enthalpy, according to literature^55,56^.

### Enantioselective analysis

The enantioselective analysis was conducted on two different GC columns, whose stationary phases were based on β-cyclodextrins (2,3-diacetyl-6-*tert*-butyldimethylsilyl-β-cyclodextrin and 2,3-diethyl-6-*tert*-butyldimethylsilyl-β-cyclodextrin), purchased from MEGA S.r.l., Legnano (MI), Italy. The columns were 25 m long, 0.25 mm internal diameter and 0.25 μm phase thickness, installed in the same GC–MS instrument described for the qualitative analysis. The analytical method was based on the following thermal program: 50 °C for 1 min, a thermal gradient of 2 °C/min until 220 °C, that were maintained for 10 min. The carrier gas (He) was set at the constant pressure of 70 kPa. Sample volumes, injector temperature, transfer line temperature, and MS parameters were the same as the qualitative analyses, whereas split ratio was 20:1 in order to significantly increase sensitivity. The enantiomers of the main chiral components were identified by mass spectrum and injection of enantiomerically pure standards (Sigma-Aldrich, St. Louis, MO, USA).

### Policy statement about plant investigation

The authors declare that all experimental research and field studies on plants (either cultivated or wild), including the collection of plant material, were carried out in accordance with relevant institutional, national, and international guidelines and legislation. Furthermore, this study was conducted under permission of the Ministry of Environment, Water and Ecological Transition of Ecuador, with MAATE registry number MAE-DNB-CM-2016–0048. Finally, the authors declare that a minimal quantity of plant material was collected to carry on the present investigation, avoiding any injury to the shrubs at the collection site.

## Conclusions

The dry leaves of *Gynoxys szyszylowiczii* Hieron. produce an EO, with a yield of 0.03% by weight. On the one hand, the chemical profile is consistent with the ones previously described for *G. laurifolia*, *G. rugulosa*, and *G. miniphylla*, but very different from *G. buxifolia*. The chemical profile showed a volatile fraction dominated by sesquiterpenes and heavy aliphatic compounds (hydrocarbons and alkenes). On the other hand, the enantiomeric composition of the *Gynoxys* spp. studied so far did not apparently present a common pattern. For a more objective interpretation of *Gynoxys* metabolism, a statistical comparison of all the EOs will be the object of a future study (Supplementary Information file 1).

### Supplementary Information

Supplementary Figures.

## Data Availability

The datasets used and/or analysed during the current study are available from the corresponding author on reasonable request.
