# Supplementary material for: A new leaf essential oil from the Andean species Gynoxys szyszylowiczii Hieron. of southern Ecuador: chemical and enantioselective analyses
Source: Sci Rep. 2024 Jul 16;14:16360. doi: 10.1038/s41598-024-67482-z (PMC11252159; doi:10.1038/s41598-024-67482-z)
Supplement: Supplementary file 1 — Supplementary Figures. [file 41598_2024_67482_MOESM1_ESM.docx]

**SUPPLEMENTARY MATERIAL**

**A New Leaf Essential Oil from the Andean Species *Gynoxys szyszylowiczii* Hieron. of Southern Ecuador: Chemical and Enantioselective Analyses**

Yessenia E. Maldonado^1^, Omar Malagón^1^, Nixon Cumbicus^2^, and Gianluca Gilardoni^1,^*

^1^ Departamento de Química, Universidad Técnica Particular de Loja (UTPL), Calle Marcelino Champagnat s/n, 110107, Loja, Ecuador.

^2^ Departamento de Ciencias Biológicas y Agropecuarias, Universidad Técnica Particular de Loja (UTPL), Calle Marcelino Champagnat s/n, Loja 110107, Ecuador.

* Corresponding author: [gianluca.gilardoni@gmail.com](mailto:gianluca.gilardoni@gmail.com)

**Contents:** Figure S1. Total ion current (TIC) enantioselective separation of partially resolved α-pinene enantiomers on a 2,3-diacetyl-6-*tert*-butyldimethylsilyl-β-cyclodextrin stationary phase; Figure S2. Total ion current (TIC) enantioselective separation of partially resolved α-pinene enantiomers on a 2,3-diethyl-6-*tert*-butyldimethylsilyl-β-cyclodextrin stationary phase; Figure S3. Single ion current (extracted ions 71, 121 *m/z*) enantioselective separation of resolved linalool enantiomers on a 2,3-diacetyl-6-*tert*-butyldimethylsilyl-β-cyclodextrin stationary phase; Figure S4. Single ion current (extracted ions 71, 111, 154 *m/z*) enantioselective separation of resolved terpinene-4-ol enantiomers on a 2,3-diacetyl-6-*tert*-butyldimethylsilyl-β-cyclodextrin stationary phase; Figure S5. Single ion current (extracted ions 133, 147 *m/z*) enantioselective separation of resolved germacrene D enantiomers on a 2,3-diethyl-6-*tert*-butyldimethylsilyl-β-cyclodextrin stationary phase.

(1*R*,5*R*)-(+)-α-pinene

(1*S*,5*S*)-(-)-α-pinene

**Figure S1.** Total ion current (TIC) enantioselective separation of partially resolved α-pinene enantiomers on a 2,3-diacetyl-6-*tert*-butyldimethylsilyl-β-cyclodextrin stationary phase.

(1*R*,5*R*)-(+)-β-pinene

(1*S*,5*S*)-(-)-β-pinene

**Figure S2.** Total ion current (TIC) enantioselective separation of partially resolved α-pinene enantiomers on a 2,3-diethyl-6-*tert*-butyldimethylsilyl-β-cyclodextrin stationary phase.

(*S*)-(+)-linalool

(*R*)-(-)-linalool

**Figure S3.** Single ion current (extracted ions 71, 121 *m/z*) enantioselective separation of resolved linalool enantiomers on a 2,3-diacetyl-6-*tert*-butyldimethylsilyl-β-cyclodextrin stationary phase.

(*S*)-(+)-terpinen-4-ol

(*R*)-(-)-terpinen-4-ol

**Figure S4.** Single ion current (extracted ions 71, 111, 154 *m/z*) enantioselective separation of resolved terpinene-4-ol enantiomers on a 2,3-diacetyl-6-*tert*-butyldimethylsilyl-β-cyclodextrin stationary phase.

(*S*)-(-)-germacrene D

(*R*)-(+)-germacrene D

**Figure S5.** Single ion current (extracted ions 133, 147 *m/z*) enantioselective separation of resolved germacrene D enantiomers on a 2,3-diethyl-6-*tert*-butyldimethylsilyl-β-cyclodextrin stationary phase.
